# Incidência de Complicações Cardiovasculares em Pacientes Pediátricos Tratados com Antraciclinas em um Centro Oncológico Brasileiro

**DOI:** 10.36660/abc.20210352

**Published:** 2024-06-06

**Authors:** Cristina Chaves dos Santos de Guerra, Geisa Sant'Ana, Osório Luiz Rangel de Almeida

**Affiliations:** 1 Hospital da Criança de Brasília José de Alencar Brasília DF Brasil Hospital da Criança de Brasília José de Alencar, Brasília, DF – Brasil; 2 Escola Superior em Ciências da Saúde Fundação de Ensino e Pesquisa em Ciências da Saúde Secretaria de Estado da Saúde do Distrito Federal Brasília DF Brasil Escola Superior em Ciências da Saúde – Fundação de Ensino e Pesquisa em Ciências da Saúde – Secretaria de Estado da Saúde do Distrito Federal, Brasília, DF – Brasil

**Keywords:** Cardiotoxicidade, Antraciclinas, Neoplasias, Criança, Tratamento Farmacológico

## Abstract

**Fundamento:**

A introdução das antraciclinas no tratamento do câncer infantojuvenil propiciou um aumento significativo na sobrevida, mas também nas taxas de morbimortalidade devido às complicações cardiovasculares (CVs).

**Objetivos:**

Conhecer o perfil cardiológico de pacientes pediátricos tratados com antraciclinas em um centro oncológico no Brasil e a incidência das complicações CVs.

**Métodos:**

Foram coletados, de prontuários de pacientes de ambos os sexos com idade até 19 anos – frequência e forma de apresentação clínica das complicações CVs Gerais (G1) e relacionadas à Disfunção Ventricular (G2) – e correlacionados com fatores de risco, faixa etária e estado vital, medicações cardiológicas e cardioprotetoras. Um valor de p < 0,05 foi considerado significativo.

**Resultados:**

Foram incluídos 326 pacientes, destes, 214 (65,6%) eram menores de 10 anos e 192 (58,89%) do sexo masculino. As complicações do G1 ocorreram em 141 (43,3%) pacientes e a mais frequente foi a hipertensão arterial sistêmica; as complicações do G2 ocorreram em 84 pacientes (25,76%). Uma Dose Cumulativa (DC) das antraciclinas > 250mg/m^2^ foi usada em 26,7% dos pacientes e a associação de complicações do G2 com essa DC não mostrou significância estatística (p=0,305; RC=1,330 e [95% IC= 0,770- 2,296]). As medicações cardiológicas mais usadas foram os diuréticos em 34,7% dos pacientes.

**Conclusões:**

O estudo mostrou, como na literatura, uma alta incidência de complicações CVs no tratamento do câncer infantojuvenil, sendo as do G1 as mais frequentes.

## Introdução

Nas últimas décadas, a taxa de sobrevida após o tratamento do câncer infantojuvenil aumentou consideravelmente, cerca de 80%, principalmente pela introdução de novos protocolos terapêuticos.^1,2^ Contudo, devido aos efeitos adversos sobre o sistema cardiovascular (CV), houve um aumento na morbimortalidade de 8,4 vezes em sobreviventes.^2,3^

As complicações CVs podem levar a, pelo menos, uma internação em até 8,1% dos casos após o tratamento,^1^ com taxa de hospitalização 14 vezes maior em sobreviventes na primeira década de vida, comparada com adultos de 60 anos.^1^Essas complicações podem ser causadas por diferentes grupos de quimioterápicos e ter diferentes formas de apresentação.^1,2^ As alterações relacionadas à disfunção cardíaca estão mais frequentemente ligadas à toxicidade das antraciclinas ao cardiomiócito e à microvasculatura.^4,5^ Ela é causada principalmente pelo estresse oxidativo, com a formação do complexo ferro-antraciclina intracelular responsável pela geração dos radicais superóxidos e pela ação sobre a enzima topoisomerase 2β, sinalizando a necrose e a apoptose celular.^4^ O dexrazoxano, é um quelante de ferro que inibe a formação desses complexos quando administrado antes de cada dose de antraciclina, minimizando os efeitos cardiotóxicos, e é empregado com função cardioprotetora.^4–7^

A presença de disfunções cardíacas associadas ao quadro de insuficiência cardíaca foi descrita, pela primeira vez, como um efeito adverso das antraciclinas, em 1967, e a relação com a dose, em 1971, podendo se manifestar logo após a exposição ou anos depois do tratamento.^2,3,5,8,9^ Os estudos com análise retrospectiva de efeitos tardios em sobreviventes de tratamento de câncer foram as principais fontes para o conhecimento atual sobre a cardiotoxicidade dessas medicações.^8,9^

No Brasil, embora haja vários centros pediátricos de tratamento oncológico, ainda carecemos de dados estatísticos mais robustos sobre a incidência de complicações CVs nesta população. O objetivo deste estudo é conhecer o perfil cardiológico de pacientes oncológicos pediátricos que usaram antraciclinas em um centro oncológico brasileiro, identificando a frequência dessas alterações, os fatores de risco, e as formas clínicas de apresentação, para que essas informações possam auxiliar na formulação de futuras estratégias de prevenção e redução de danos.

## Métodos

### Delineamento do estudo

Este foi um estudo observacional, longitudinal, retrospectivo, descritivo e analítico, realizado por meio de pesquisa em prontuários físicos e eletrônicos. Realizou-se coleta de dados desde o início do tratamento dos pacientes, com os seguintes critérios de inclusão: ambos os sexos, com idade até 19 anos, portadores de doença neoplásica tratados com antraciclinas, com início de tratamento entre 2014 e 2018 e conclusão até 10 de abril de 2020. Os critérios de exclusão foram informações incompletas.

Esta pesquisa foi desenvolvida após aprovação pelo Comitê de Ética em Pesquisas da nossa instituição conforme protocolo n^o^ 3.711.502.

### Variáveis do estudo e coleta de dados

Foram consideradas as seguintes variáveis: idade ao diagnóstico; sexo; procedência; tipo de câncer e sua distribuição por faixa etária; complicações CV, uso de medicações cardiológicas e de dexrazoxano; relação das complicações CV com idade e estado vital (vivo/óbito); causas dos óbitos e avaliação cardiológica.

As complicações CVs foram divididas em dois grupos: Gerais (G1) e Disfunção Ventricular (G2). As complicações do G1 foram Hipertensão Arterial Sistêmica (HAS); derrame pericárdico; Fenômenos Tromboembólicos (FTE) venosos; alterações do ritmo; miocardite; endocardite; isquemia ou Infarto Agudo do Miocárdio (IAM); acidente vascular cerebral (AVC) e insuficiência cardíaca congestiva (ICC) por causas não relacionadas às antraciclinas.^4,5,10^ As complicações G2 foram cardiotoxicidade suspeita, determinada pela queda da Fração de Ejeção do Ventrículo Esquerdo (FEVE) em 10 pontos em relação ao ecocardiograma basal e maior ou igual a 55%; disfunção diastólica de ventrículo direito (DD-VD); disfunção diastólica do ventrículo esquerdo (DD-VE); disfunção sistólica do VE (DS-VE) quando FEVE < 55% e percentual de encurtamento sistólico (%D < 28%).^4,5^ A % D e a FEVE foram avaliadas pelo ecocardiograma convencional, pelo modo M com método de Teichholz; e a função diastólica foi avaliada pelo Doppler pulsado e tecidual e pela análise do diâmetros do átrio esquerdo, por mais de um observador.

Os fatores de risco para cardiotoxicidade relacionados ao G2 avaliados foram idade; sexo feminino; Dose Cumulativa (DC) de antraciclinas maior que 250mg/m^2^; associação com outras drogas cardiotóxicas (ifosfamida e ciclofosfamida), radioterapia mediastinal ou torácica; presença de síndrome genética, e presença de cardiopatia congênita. A DC das antraciclinas foi convertida em doxorrubicina-equivalência.^11^

### Análise estatística

Foram feitas análises descritivas e de associação. As variáveis qualitativas nominais e ordinais foram apresentadas por meio de frequência (n) e porcentagem (%). Foram avaliadas associações das complicações CVs relacionadas à faixa etária e ao estado vital; a média da idade ao diagnóstico foi descrita por média e desvio-padrão. As análises foram feitas por meio do teste qui-quadrado de Pearson com correção de continuidade (estado vital) e simulação de Monte Carlo (para faixa etária) quando necessário (ao menos uma célula tinha uma frequência esperada menor que 5). Para o estado vital, foi possível calcular a razão de chance para variáveis com duas categorias e na ausência de células iguais a zero. As análises dos dados foram realizadas no programa IBM SPSS (*Statistical Package for the Social Sciences*) 23, de 2015. O nível de significância utilizado em todo o estudo foi de 5%.

## Resultados

Dos 826 pacientes admitidos, 444 (53,7%) usaram antraciclinas e, desses, 326 (73,4%) foram incluídos. A procedência foi determinada em 302 (92,6%) pacientes, sendo 155 (47,5%) residentes no Distrito Federal; a média da idade ao diagnóstico foi de 6,8 anos com desvio-padrão de 5,0 anos e houve predomínio do sexo masculino com 192 (58,9 %). A Tabela 1 resume os tipos de câncer por faixa etária.

**Tabela 1:** Tipo de câncer em diferentes faixas etárias

			Faixa etária ao diagnóstico					Total
			< 1 ano	1 a 4 anos	5 a 9 anos	10 a 14 anos	15 a 19 anos	
Tipo	Leucemias	n (%)	9 (39,13)	77 (63,64)	36 (51,43)	34 (39,08)	11 (44,00)	167 (51,23)
	Linfomas	n (%)	0 (00,00)	9 (7,44)	21 (30,00)	18 (20,69)	9 (36,00)	57 (17,48)
	Tumor Renal	n (%)	4 (17,39)	16 (13,22)	4 (5,71)	0 (0,00)	0 (0,00)	24 (7,36)
	Neuroblastomas	n (%)	8 (34,78)	11 (9,09)	1 (1,43)	2 (2,30)	0 (0,00)	22 (6,75)
	Tumores hepáticos	n (%)	0 (0,00)	3 (2,48)	2 (2,86)	2 (2,30)	0 (0,00)	7 (2,15)
	Osteossarcomas	n (%)	0 (0,00)	1 (0,83)	1 (1,43)	15 (17,24)	4 (16,00)	21 (6,44)
	Sarcomas	n (%)	2 (8,70)	4 (3,31)	5 (7,14)	12 (13,79)	1 (4,00)	24 (7,36)
	Outros	n (%)	0 (0,00)	0 (0,00)	0 (0,00)	4 (4,60)	0 (0,00)	4 (1,23)
Total		n	23	121	70	87	25	326
		%	100	100	100	100	100	100

### Complicações CV do G1

As complicações CVs do G1 ocorreram em 141 (43,3%) pacientes, sendo que em alguns houve mais de uma complicação. A HAS ocorreu em 50 (15,3%); derrame pericárdico em 48 (14,7%); FTE em 41 (12,57%); alteração do ritmo em 32 (9,8%); ICC com uso de drogas vasoativas em 25 (7,7%); miocardite em 4 (1,22%); endocardite em 4 (1,2%); isquemia com IAM em 1 (0,3%) e AVC em 1 (0,3%).

### Complicações CV do G2 e associação com fatores de risco para cardiotoxicidade

As complicações CV do G2 ocorreram em 84 (25,8%) pacientes, sendo que em alguns houve mais de uma complicação. A suspeita da cardiotoxicidade ocorreu em 49 (15,0%); a DD-VD em 15 (4,6%); a DD-VE em 36 (11,0%); e a DS-VE em 24 (7,4%). Não se observou associação significativamente entre os fatores de risco para cardiotoxicidade e complicações do G2 (Tabela 2).

**Tabela 2:** Associação dos fatores de risco para cardiotoxicidade com complicações cardiovasculares do grupo G2 (disfunção ventricular)

		Complicação cardiovascular do G2		Total n (%)	p	RC	IC 95%
		Não n (%)	Sim n (%)				
< 5 anos	Não	128 (52,89)	54 (64,29)	182 (55,83)	0,070	0,624	0,374 - 1,042
	Sim	114 (47,11)	30 (35,71)	144 (44,17)			
Sexo feminino	Masculino	141 (58,26)	51 (60,71)	192 (58,90)	0,694	0,903	0,544 - 1,500
	Feminino	101 (41,74)	33 (39,29)	134 (41,10)			
Síndrome Genética	Sim - S Down	8 (3,31)	1 (1,19)	9 (2,76)	0,561	-	-
	Sim - Outra	11 (4,55)	3 (3,57)	14 (4,29)			
	Não	223 (92,15)	80 (95,24)	303 (92,94)			
Cardiopatia prévia	Sim	11 (4,55)	3 (3,57)	14 (4,29)	0,947	1,286	0,350 - 4,724
	Não	231 (95,45)	81 (96,43)	312 (95,71)			
DC de antraciclinas	Dose até 249	181 (74,79)	58 (69,05)	239 (73,31)	0,305	1,330	0,770 - 2,296
	Dose > 250	61 (25,21)	26 (30,95)	87 (26,69)			
Radioterapia	Sim	19 (7,85)	5 (5,95)	24 (7,36)	0,566	1,346	0,486 - 3,726
Mediastinal/tórax	Não	223 (92,15)	79 (94,05)	302 (92,64)			
Outras Drogas cardiotóxicas	Sim	147 (60,74)	61 (72,62)	208 (63,80)	0,051	0,583	0,338 - 1,006
	Não	95 (39,26)	23 (27,38)	118 (36,20)			
**TOTAL**		242 (100)	84 (100)	326 (100)			

DC: dose cumulativa; RC: risco de chance (Oddis ratio); IC: intervalo de confiança.

### Uso de drogas cardiológicas e de Dexrazoxano

As drogas cardiológicas mais utilizadas foram: diuréticos por 113 (34,7%) pacientes, anti-hipertensivos por 56 (17,2%) e drogas vasoativas por 54 (16,6%).

Dos 73 pacientes que usaram Dexrazoxano, 18 (24,7%) apresentaram as complicações do G2, e somente 36 (49,3%) usaram o cardioprotetor em 100% das doses de antraciclinas. Dos 253 pacientes que não usaram, 66 (26,08%) apresentaram complicações do G2. O uso da Dexrazoxano não mostrou associação significante com cardioproteção (p=0,806; RC=1,078 e [95% I.C.= 0,591-1,968]).

### Complicações CVs do G1 e G2 e associação com faixa etária e estado vital

As complicações CV dos G1 e G2 ocorreram em 173 (53,1%) pacientes. A Tabela 3 resume a associação dessas complicações e a faixa etária ao diagnóstico, e a Tabela 4, a associação entre as complicações do G1 e G2 com estado vital

**Tabela 3:** Análise de associação das complicações cardiovasculares gerais (G1) e disfunção ventricular (G2) com faixa etária ao diagnóstico

		Faixa etária					Total	p*
		< 1 ano n (%)	1-4 anos n (%)	5-9 anos n (%)	10-14 anos n (%)	15-19anos n (%)		
Alterações do ritmo	Não	21 (91,3)	109 (90,1)	65 (92,9)	76 (87,4)	23 (92,0)	294 (90,2)	0,839
	Sim	2 (8,7)	12 (9,9)	5 (7,1)	11 (12,6)	2 (8,0)	32 (9,8)	
Miocardite	Não	23 (100,0)	119 (98,3)	69 (98,6)	87 (100,0)	24 (96,0)	322 (98,8)	0,553
	Sim	0 (0,0)	2 (1,7)	1 (1,4)	0 (0,0)	1 (4,0)	4 (1,2)	
Isquemia- IAM	Não	23 (100,0)	121 (100,0)	70 (100,0)	87 (100,0)	24 (96,0)	325 (99,7)	0,143
	Sim	0 (0,0)	0 (0,0)	0 (0,0)	0 (0,0)	1 (4,0)	1 (0,3)	
HAS	Não	15 (65,2)	104 (85,9)	58 (82,9)	77 (88,5)	22 (88,0)	276 (84,7)	0,080
	Sim	8 (34,8)	17 (14,1)	12 (17,1)	10 (11,5)	3 (12,0)	50 (15,3)	
FTE com cateter	Não	23 (100,0)	118 (97,5)	66 (94,3)	83 (95,4)	24 (96,0)	314 (96,3)	0,696
	Sim	0 (0,00)	3 (2,5)	4 (5,7)	4 (4,6)	1 (4,0)	12 (3,7)	
FTE sem cateter	Não	21 (91,3)	113 (93,4)	63 (90,0)	79 (90,8)	21 (84,0)	297 (91,1)	0,672
	Sim	2 (8,7)	8 (6,6)	7 (10,0)	8 (9,2)	4 (16,0)	29 (8,9)	
DP sem antraciclinas	Não	22 (95,6)	113 (93,4)	64 (91,4)	82 (94,3)	20 (80,0)	301 (92,3)	0,163
	Sim	1 (4,4)	8 (6,6)	6 (8,6)	5 (5,7)	5 (20,0)	25 (7,7)	
DP com antraciclinas	Não	22 (95,6)	112 (92,6)	69 (98,6)	81 (93,1)	23 (92,0)	307 (94,2)	0,481
	Sim	1 (4,4)	9 (7,4)	1 (1,4)	6 (6,9)	2 (8,0)	19 (5,8)	
DP com drenagem	Não	22 (95,6)	121 (100,0)	70 (100,0)	84 (96,5)	25 (100,0)	322 (98,8)	0,073
	Sim	1 (4,4)	0 (0,0)	0 (0,0)	3 (3,5)	0 (0,0)	4 (1,2)	
AVC	Não	22 (95,6)	121 (100,0)	70 (100,0)	87 (100,0)	25 (100,0)	325 (99,7)	0,069
	Sim	1 (4,5)	0 (0,0)	0 (0,0)	0 (0,0)	0 (0,0)	1 (0,3)	
ICC com DVA	Não	17 (73,9)	113 (93,4)	66 (94,3)	82 (94,3)	23 (92,0)	301 (92,3)	0,019
	Sim	6 (26,1)	8 (6,6)	4 (5,7)	5 (5,7)	2 (8,0)	25 (7,7)	
Endocardite	Não	22 (95,6)	120 (99,2)	69 (98,6)	86 (98,8)	25 (100,0)	322 (98,8)	0,740
	Sim	1 (4,4)	1 (0,8)	1 (1,4)	1 (1,2)	0 (0,0)	4 (1,2)	
DD-VD	Não	22 (95,6)	117 (96,7)	68 (97,1)	81 (93,1)	23 (92,0)	311 (95,4)	0,622
	Sim	1 (4,4)	4 (3,3)	2 (2,9)	6 (6,9)	2 (8,0)	15 (4,6)	
Cardiotoxicidade suspeita	Não	19 (82,6)	108 (89,3)	56 (80,0)	74 (85,1)	20 (80,0)	277 (85,0)	0,458
	Sim	4 (17,4)	13 (10,7)	14 (20,0)	13 (14,9)	5 (20,0)	49 (15,0)	
DD-VE	Não	20 (87,0)	110 (90,9)	63 (90,0)	74 (85,1)	23 (92,0)	290 (89,0)	
	Sim DC=0	1 (4,3)	3 (2,5)	0 (0,0)	6 (6,9)	1 (4,0)	11 (3,4)	0,766
	Sim DC<250	2 (8,7)	6 (5,0)	6 (8,6)	6 (6,9)	1 (4,0)	21 (6,4)	
	Sim DC>250	0 (0,0)	2 (1,6)	1 (1,4)	1 (1,1)	0 (0,0)	4 (1,2)	
DS-VE	Não	23 (100,0)	118 (97,5)	63 (90,0)	78 (89,6)	20 (80,0)	302 (92,7)	
	Sim DC=0	0 (00,0)	0 (00,00)	0 (00,0)	4 (4,6)	0 (00,0)	4 (1,2)	0,009
	Sim DC<250	0 (00,0)	1 (0,8)	6 (8,6)	4 (4,6)	3 (12,0)	14 (4,3)	
	Sim DC>250	0 (0,0)	2 (1,7)	1 (1,4)	1 (1,2)	2 (8,0)	6 (1,8)	
**TOTAL**		23 (100)	121 (100)	70 (100)	87 (100)	25 (100)	326 (100)	

IAM: infarto agudo do miocárdio; HAS: hipertensão arterial sistêmica; FTE: fenômenos tromboembólicos; DP: derrame pericárdico; AVC: acidente vascular cerebral; ICC com DVA: insuficiência cardíaca com uso de drogas vasoativas; DD-VD: disfunção diastólica de ventrículo direito; DD-VE: disfunção diastólica de ventrículo esquerdo; DS-VE: disfunção sistólica de ventrículo esquerdo, DC: dose cumulativa;

* Nível de significância de 5%.

**Tabela 4:** Análise de associação das complicações cardiovasculares gerais (G1) e disfunção ventricular (G2) com estado vital

		Estado Vital		Óbito n (%)	p*	RC	IC 95%
		Total n (%)	Vivo n (%)				
Alterações do ritmo	Não	210 (89,36)	84 (92,31)	294 (90,18)	0,423	0,700	0,292 - 1,680
	Sim	25 (10,64)	7 (7,69)	32 (9,82)			
Miocardite	Não	234 (99,57)	88 (96,70)	322 (98,77)	0,121	7,977	0,819 - 77,711
	Sim	1 (0,43)	3 (3,30)	4 (1,23)			
Isquemia- IAM	Não	235 (100,00)	90 (98,90)	325 (99,69)	0,622	-	-
	Sim	0 (0,00)	1 (1,10)	1 (0,31)			
HAS	Não	210 (89,36)	66 (72,53)	276 (84,66)	<0,001	3,182	1,712 - 5,912
	Sim	25 (10,64)	25 (27,47)	50 (15,34)			
FTE com cateter	Não	227 (96,60)	87 (95,60)	314 (96,32)	0,921	1,305	0,383 - 4,443
	Sim	8 (3,40)	4 (4,40)	12 (3,68)			
FTE sem cateter	Não	216 (91,91)	81 (89,01)	297 (91,10)	0,409	1,404	0,626 - 3,146
	Sim	19 (8,09)	10 (10,99)	29 (8,90)			
DP sem antraciclinas	Não	220 (93,62)	81 (89,01)	301 (92,33)	0,161	1,811	0,782 - 4,193
	Sim	15 (6,38)	10 (10,99)	25 (7,67)			
DP com antraciclinas	Não	226 (96,17)	81 (89,01)	307 (94,17)	0,013	3,100	1,216 - 7,902
	Sim	9 (3,83)	10 (10,99)	19 (5,83)			
DP com drenagem	Não	234 (99,57)	88 (96,70)	322 (98,77)	0,121	7,977	0,819 - 77,711
	Sim	1 (0,43)	3 (3,30)	4 (1,23)			
AVC	Não	234 (99,57)	91 (100,00)	325 (99,69)	1,000	-	-
	Sim	1 (0,43)	0 (0,00)	1 (0,31)			
ICC com DVA	Não	225 (95,74)	76 (83,52)	301 (92,33)	<0,001	4,441	1,915 - 10,300
	Sim	10 (4,26)	15 (16,48)	25 (7,67)			
Endocardite	Não	232 (98,72)	90 (98,90)	322 (98,77)	1,000	0,859	0,088 - 8,369
	Sim	3 (1,28)	1 (1,10)	4 (1,23)			
DD-VD	Não	227 (96,60)	84 (92,31)	311 (95,40)	0,173	2,365	0,832 - 6,722
	Sim	8 (3,40)	7 (7,69)	15 (4,60)			
Cardiotoxicidade subclínica	Não	195 (82,98)	82 (90,11)	277 (84,97)	0,106	0,535	0,248 - 1,153
	Sim	40 (17,02)	9 (9,89)	49 (15,03)			
DD-VE	Não	211 (89,79)	79 (86,81)	290 (88,96)	0,676	-	-
	Sim DC=0	6 (2,55)	5 (5,49)	11 (3,37)			
	Sim DC<250	15 (6,38)	6 (6,59)	21 (6,44)			
	Sim DC>250	3 (1,28)	1 (1,10)	4 (1,23)			
DS-VE	Não	219 (93,19)	83 (91,21)	302 (92,64)	0,757	-	-
	Sim DC=0	3 (1,28)	1 (1,10)	4 (1,23)			
	Sim DC<250	10 (4,26)	4 (4,40)	14 (4,29)			
	Sim DC>250	3 (1,28)	3 (3,30)	6 (1,84)			
**TOTAL**	235 (100)	91 (100)	326 (100)				

IAM: infarto agudo do miocárdio; HAS: hipertensão arterial sistêmica; FTE: fenômenos tromboembólicos; DP: derrame pericárdico; AVC: acidente vascular cerebral; ICC com DVA: insuficiência cardíaca com uso de drogas vasoativas; DD-VD: disfunção diastólica de ventrículo direito; DD-VE: disfunção diastólica de ventrículo esquerdo; DS-VE: disfunção sistólica de ventrículo esquerdo; DC: dose cumulativa;

* Nível de significância de 5%.

### Causas de óbitos

Foram a óbito 91 (27,91%) pacientes. O tempo médio até o óbito foi 17 meses, as causas foram recorrência e/ou progressão da doença em 57 (62,6%) pacientes, doenças infecciosas e/ou parasitárias em 16 (17,6%), insuficiência respiratória aguda em oito (8,8%), doença CV em cinco (5,5%), e outras em cinco (5,5%).

### Avaliação cardiológica

**Figura 1:**
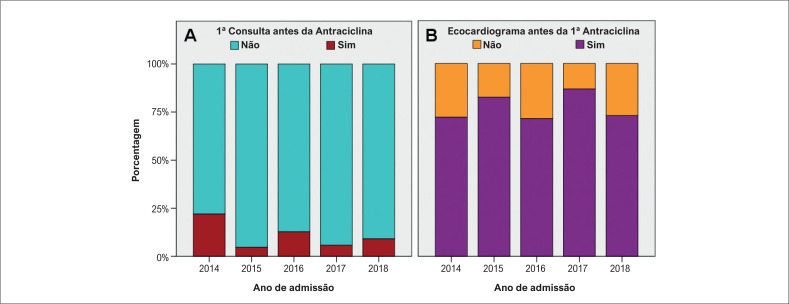
A) Primeira consulta x primeira dose de antraciclinas. B) Primeiro ecocardiograma x primeira dose de antraciclinas.

Realizaram consulta cardiológica pediátrica 216 pacientes (66.3%) e 318 ecocardiograma (97,54%). A Figura 1A e B resume a porcentagem de pacientes que compareceram na primeira consulta e que realizaram o ecocardiograma antes da primeira dose de antraciclinas.

## Discussão

As antraciclinas são empregadas em mais de 50% dos tratamentos de câncer infantojuvenil.^12^

Segundo o Ministério da Saúde, existem 317 unidades habilitadas para o tratamento de câncer no Brasil;^13^ mesmo assim, aproximadamente 50% dos pacientes do estudo não residiam no Distrito Federal.

Em concordância com a literatura, houve predominância em crianças de até quatro anos de idade e do sexo masculino (Tabela 1).^14–16^ Além disso, predominaram as leucemias (Tabela 1) que correspondem a 33% de todos os cânceres na faixa etária entre 0 e 14 anos.^17,18^

Durante e após o tratamento, diversas complicações CV podem ocorrer, com diferentes formas de apresentação clínica e gravidade^3,10,19^(Tabelas 3 e 4), podendo ocorrer antes do uso das antraciclinas nas leucemias.^20,21^

Segundo a literatura, durante o tratamento do câncer infantojuvenil, as alterações CVs são as complicações mais frequentes não relacionadas ao tumor, que podem contribuir para uma maior morbimortalidade precoce na vida adulta. Apesar disso, não dispomos de dados estatísticos robustos da incidência dessas complicações nessa população em território brasileiro, podendo dar/ a impressão de que essas alterações não estão presentes em nossa realidade. A pesquisa constatou, como descrito na literatura, que as complicações CVs foram frequentes, mas, para nossa surpresa, as mais prevalentes foram as não relacionadas à disfunção ventricular. Assim, essas formas de apresentação devem ser consideradas na formulação das estratégias de prevenção.

As Tabelas 3 e 4 resumem a frequência das complicações CVs, sendo a HAS a mais prevalente, provavelmente relacionada ao emprego de glicocorticoides na fase de indução de alguns protocolos, e isso pode explicar por que as drogas mais usadas foram os diuréticos e anti-hipertensivos.^22^ O derrame pericárdico pode ocorrer em até 21% dos pacientes;^4^ no estudo, essa foi a segunda complicação CV mais comum, ocorrendo mesmo antes do uso das antraciclinas. Os FTE venosos corresponderam à terceira complicação CV mais frequente, podendo ocorrer pela presença de acesso venoso de longa permanência ou pelo efeito trombogênico das células tumorais, com incidência de até 20% em pacientes adultos hospitalizados e 8% nas crianças.^3–5^ A incidência das alterações do ritmo cardíaco pode estar subestimada devido a não realização de método diagnóstico de rotina; a incidência descrita é de até 38%.^4^ A ICC por outras causas, com uso de drogas vasoativas, ocupou o quinto lugar no estudo, e isso pode ocorrer devido à sobrecarga volêmica, imunossupressão que favorece quadros infecciosos com disfunção miocárdica transitória e/ou choque séptico.^23^

O dano miocárdico subclínico pode ocorrer com a FEVE e a % D ainda normais que, quando alteradas, esse dano já seria irreversível.^5,24–27^ Com a intenção de aplicar métodos mais sensíveis, novas técnicas vêm sendo empregadas, entre elas está o Strain Longitudinal Global Longitudinal (SLG) para diagnóstico de disfunção subclínica com alta sensibilidade.^5,24–27^

No estudo, as complicações CV ocorreram em mais de 50% dos pacientes, e sabemos que dois de cada três sobreviventes podem ter alguma complicação cardiovascular até 30 anos após o tratamento oncológico.^4,28^

Na avaliação da associação dos fatores de risco com complicações CV do G2 (Tabela 2), a DC> 250mg/m^2^ não demonstrou significância, apesar de esse ser o principal fator de risco para cardiotoxicidade do G2^3,29^e de quase 2/3 dos pacientes terem utilizado DC< 250mg/m^2^. As complicações CV do G2 não foram infrequentes (Tabelas 3 e 4), demonstrando que não existe dose segura, como também foi evidenciada a presença de alterações subclínicas pelo ecocardiograma com dose de antraciclinas de 100 mg/m^2^.^2^

Em estudos controlados em que o Dexrazoxano foi utilizado em todas as doses de antraciclinas, foi demonstrada a cardioproteção em relação ao grupo controle.^2,6,7^ Em nossas observações, o uso de Dexrazoxano não demonstrou significância estatística para cardioproteção, o que pode ser explicado pelo fato de que mais de 50% dos pacientes não utilizaram o cardioprotetor em todas as doses de antraciclinas conforme recomendações, sendo este um viés do nosso trabalho.^30^

Como mostrado na Tabela 3, ICC com uso de drogas vasoativas apresentou associação significativa com idade inferior a um ano, o que pode ser explicado pela imaturidade do sistema CV e a sensibilidade relativa das células mais jovens à quimioterapia.^4^ Ainda, a DS-VE associou-se significativamente com idade maior que 15 anos, o que pode ser explicado pela predominância de tumores que usam altas DC de antraciclinas.^17^ Na Tabela 4, apresentaram associação significativa com óbito – HAS, derrame pericárdico após início das antraciclinas e ICC com DVA – contudo, concordando com a literatura, no estudo, a recorrência com progressão da doença subjacente foi a principal causa de óbito.^1^

As complicações CVs são as complicações mais frequentes relacionadas ao tratamento antineoplásico são as CV. A avaliação cardiológica desde as fases iniciais do tratamento para estratificação de risco, a aplicação de protocolos de seguimento e a implementação de medidas preventivas são de fundamental importância.^3,5^ No estudo, cerca de 80% dos pacientes realizaram ecocardiograma antes do uso de antraciclinas, o mesmo não ocorreu em relação às consultas cardiológicas (Figura 1).

Este estudo apresenta algumas limitações a serem consideradas. O desenho retrospectivo e unicêntrico expõe o viés de informação e a inabilidade para controlar variáveis de confusão, (falta de informação). Assim, informações das complicações CVs poderiam estar subestimadas ao realizar avaliação ecocardiográfica com modo M (Teichholz), sendo que as recomendações orientam para avaliação volumétrica do ventrículo esquerdo (Simpson biplanar). No período estudado, a falta de outros métodos que auxiliam no diagnóstico de alterações subclínicas da função ventricular, como o ecocardiograma com *strain*, avaliação dos biomarcadores de lesão miocárdica, assim como a realização rotineira do eletrocardiograma, pode ter subestimado a incidência das complicações do G2 e de alterações do ritmo cardíaco.

## Conclusões

Embora as complicações CVs relacionadas à disfunção ventricular sejam as mais graves, mais temidas e mais estudadas, o presente estudo mostrou que as gerais foram mais frequentes, denotando a necessidade dessas formas de apresentação serem incluídas nas estratégias de monitoramento e prevenção.

A alta DC de antraciclinas é o principal fator de risco para cardiotoxicidade relacionada à disfunção ventricular, mas não existe dose segura. O estudo reforça esse entendimento uma vez que 73,3% dos pacientes usaram DC < 250mg/m^2^ e mesmo assim essas complicações ocorreram em um de cada quatro pacientes.

Apesar das limitações, o estudo oportunizou um primeiro levantamento, já que existe uma escassez de trabalhos brasileiros publicados com análises das alterações CVs durante o tratamento quimioterápico na população infantojuvenil. Porém, o cenário clínico aqui apresentado certamente reproduz a realidade de outros centros de tratamento do câncer. Por isso, esperamos chamar a atenção para a necessidade do reconhecimento *in loco* das reais demandas, buscando instituir estratégias para o aprimoramento dos pontos fortes e o ajuste das deficiências identificadas.

Os dados encontrados neste estudo enfatizam a importância de uma parceria entre oncologistas e cardiologistas com formulação de estratégias de prevenção, diagnóstico, terapia precoce otimizada das diferentes formas de apresentação de cardiotoxicidade. Isso possibilitaria a continuidade do tratamento, uma melhor qualidade de vida futura, e uma redução das taxas de morbimortalidade.
