# Supplementary material for: Incidência de Complicações Cardiovasculares em Pacientes Pediátricos Tratados com Antraciclinas em um Centro Oncológico Brasileiro
Source: Arq Bras Cardiol. 2024 Jun 6;121(5):e20210352. [Article in Portuguese] doi: 10.36660/abc.20210352 (PMC11216333; doi:10.36660/abc.20210352)
Supplement: Translation

# Translation: Incidence of Cardiovascular Complications in Pediatric Patients Treated with Anthracyclines at a Brazilian Cancer Center

**DOI:** 10.36660/abc.20210352i

**Authors:** Cristina Chaves dos Santos de Guerra, Geisa Sant'Ana, Osório Luiz Rangel de Almeida

**Affiliations:** 1 Brasília DF Brazil Hospital da Criança de Brasília José de Alencar, Brasília, DF – Brazil; 2 Brasília DF Brazil Escola Superior em Ciências da Saúde – Fundação de Ensino e Pesquisa em Ciências da Saúde – Secretaria de Estado da Saúde do Distrito Federal, Brasília, DF – Brazil

**Keywords:** Cardiotoxicity, Anthracyclines, Neoplasms, Child, Drug Therapy

## Abstract

**Background:**

The introduction of anthracyclines in the treatment of children and adolescents with cancer has promoted a significant increase in survival, but also in morbidity and mortality rates due to cardiovascular (CV) complications.

**Objectives:**

To determine the cardiovascular profile of pediatric patients treated with anthracyclines at a cancer center in Brazil and the incidence of CV complications.

**Methods:**

The following data were collected from the medical records of patients of both sexes, aged younger than 19 years – frequency and form of clinical presentation of general CV complications (G1) and CV complications related to ventricular dysfunction (G2) – and correlated with risk factors, age range and vital status, cardiovascular and cardioprotective medications. A p<0.05 was considered statistically significant.

**Results:**

A total of 326 patients were included, 214 (65.6%) were younger than 10 years and 192 (58.9%) of male sex. G1 complications occurred in 141 (43.3%) patients, and the most frequent was systemic arterial hypertension; G2 complications occurred in 84 patients (25.8%). Cumulative dose (CD) of anthracyclines > 250mg/m^2^ was used in 26.7% of patients and the association of G2 complications with this CD was not statistically significant (p=0.305; OR=1.330 and [95% CI = 0.770- 2.296]). The most used cardiac medications were diuretics (34.7% of patients).

**Conclusions:**

In accordance with literature, the study showed a high incidence of CV complications in the treatment of children and adolescents with cancer, with general CV complications as the most prevalent.

## Introduction

In the last decades, survival rates of children and adolescents after cancer treatment have considerably increased (approximately 80¨%), mainly with the introduction of new therapeutical protocols.^1,2^ However, due to the adverse effects of the therapy on the cardiovascular (CV) system, there was an 8.4- time increase in morbidity and mortality among survivors.^2,3^

At least one hospitalization is caused by CV complications in up to 8.1% of patients in the post-treatment,^1^ and hospitalization rates are 14 times higher in survivors in the first decade of life as compared with adults older than 60 years.^1^ These complications may be caused by different chemotherapy agents and may have different presentations.^1,2^ Changes related to cardiac dysfunction are more frequently linked to anthracycline cardiotoxicity, which leads to myocardial and microvascular damage.^4,5^ It is mainly caused by oxidative stress, with formation of intracellular anthracycline-iron complexes, which are responsible for the generation of superoxide radicals, and by the effect of anthracyclines on topoisomerase 2β, thereby signaling apoptosis and necrosis.^4^ Dexrazoxane is an iron chelator that inhibits the formation of these complexes when administered prior to each dose of anthracycline, minimizing its cardiotoxic effects, and hence used with a cardioprotective function.^4–7^

The presence of cardiac dysfunction associated with heart failure was first described as an adverse event of anthracyclines in 1967, and the relationship with the dose in 1971; this may manifest either early, immediately after exposure, or late, years after treatment.^2,3,5,8,9^ Studies with retrospective analysis of late effects in cancer treatment survivors were the main source of current knowledge about cardiotoxicity of these drugs.^8,9^

In Brazil, although there are several pediatric cancer centers, there are still few statistical data on the incidence of CV complications in this population. The aim of this study was to describe the CV profile of pediatric oncologic patients treated with anthracyclines at a cancer center, identifying the frequency of these complications, risk factors, and clinical presentations. This information would help in the development of strategies for prevention and reduction of damage.

## Methods

### Study design

This was an observational, longitudinal, retrospective, descriptive and analytical study, with review of electronic and paper medical records. Data were collected from the beginning of treatment; the following inclusion criteria were used: patients of both sexes, aged less than 19 years, with neoplastic disease and treated with anthracyclines, with the onset of treatment between 2014 and 2018 and end of treatment before April 10, 2020. Patients with missing data were excluded.

The study was approved by the ethics committee of our institution (approval number 3.711.502).

### Variables and data collection

The following variables were considered for analysis: age at diagnosis, sex, place of origin, type of cancer and its distribution by age range, CV complications, use of cardiovascular medications, use of dexrazoxane, relationship of CV complications with age and vital status (alive/dead), cause of death, and cardiac assessment.

CV complications were divided into two groups – general complications (G1) and ventricular dysfunction (G2). G1 were systemic arterial hypertension (SAH), pericardial effusion, venous thromboembolic events (TEE), rhythm disturbances, myocarditis, endocarditis, ischemia or acute myocardial infarction (AMI), cerebrovascular accident (CVA), and congestive heart failure (CHF) due to causes unrelated to anthracyclines.^4,5,10^ G2 were defined as suspected cardiotoxicity, determined by a reduction in the left ventricular ejection fraction (LVEF) by 10 points in comparison with baseline echocardiogram and greater than or equal to 55%; right ventricular diastolic dysfunction (RVDD); left ventricular diastolic dysfunction (LVDD); left ventricular systolic dysfunction (LVSD) with LVEF < 55% and systolic fractional shortening (FS < 28%).^4,5^ Both FS and LVEF were assessed by conventional M-mode echocardiography, using the Teichholz formula; and diastolic function was assessed by pulsed tissue Doppler imaging, by analysis of left atrial diameter, made by two observers.

The following risk factors for cardiotoxicity, related to G2, were evaluated: age, female sex, cumulative dose (CD) of anthracyclines greater than 250mg/m^2^, association with other cardiotoxic drugs (iphosphamide and cyclophosphamide), mediastinal or thoracic radiotherapy, presence of genetic syndrome, and presence of congenital heart disease. The CD of anthracyclines was converted into doxorubicin equivalent.^11^

### Statistical analysis

Descriptive and association analyses were performed. Qualitative nominal and ordinal variables were described as frequency (n) and percentage (%). Associations of CV complications related to age and vital status were assessed; mean age at diagnosis was described as mean and standard deviation. For these analyses, Pearson's chi-square test with continuity correction (vital status) was used, as well as Monte Carlo simulation (for age range), as appropriate (at least one cell had an expected cell count less than 5). For vital status, odds ratio was calculated for variables with two categories and in the absence of zero cell count. Statistical analysis was performed using the IBM SPSS (*Statistical Package for the Social Sciences*) software 23 (2015). Statistical analysis was set at 5%.

## Results

Of the 826 patients admitted, 444 (53.7%) used anthracyclines and 326 of them (73.4%) were included. The place of origin of 302 patients (92.6%) was found in the medical records; 155 (47,5%) lived in the Federal District. Mean age at diagnosis was 6.85 ± 5.0 years, and most patients were men (n=192, 58.9%). Table 1 summarizes the types of cancer by age range.

**Table 1:** Type of cancer in different age ranges

			Age at diagnosis					Total
			< 1 year	1 – 4 years	5 – 9 years	10 – 14 years	15 – 19 years	
Type	Leukemias	n (%)	9 (39.13)	77 (63.64)	36 (51.43)	34 (39.08)	11 (44.00)	167 (51.23)
	Lymphomas	n (%)	0 (00.00)	9 (7.44)	21 (30.00)	18 (20.69)	9 (36.00)	57 (17.48)
	Renal tumor	n (%)	4 (17.39)	16 (13.22)	4 (5.71)	0 (0.00)	0 (0.00)	24 (7.36)
	Neuroblastomas	n (%)	8 (34.78)	11 (9.09)	1 (1.43)	2 (2.30)	0 (0.00)	22 (6.75)
	Liver tumor	n (%)	0 (0.00)	3 (2.48)	2 (2.86)	2 (2.30)	0 (0.00)	7 (2.15)
	Osteosarcomas	n (%)	0 (0.00)	1 (0.83)	1 (1.43)	15 (17.24)	4 (16.00)	21 (6.44)
	Sarcomas	n (%)	2 (8.70)	4 (3.31)	5 (7.14)	12 (13.79)	1 (4.00)	24 (7.36)
	Others	n (%)	0 (0.00)	0 (0.00)	0 (0.00)	4 (4.60)	0 (0.00)	4 (1.23)
Total		n	23	121	70	87	25	326
		%	100	100	100	100	100	100

### CV complications in the G1 group

In the G1 group, CV complications occurred in 141 (43.3%) patients, with more than one complication in some of them. SAH was observed in 50 patients (15.3%); pericardial effusion in 48 (14.7%); TEE in 41 (12.6%); abnormal heart rhythm in 32 (9.8%); CHF in 25 (7.7%) in patients using vasoactive drugs; myocarditis in four (1.2%); endocarditis in four (1.2%); ischemia with AMI in one (0.3%) and CVA in one (0.3%).

### CV complications related to G2 (ventricular dysfunction) and their association with risk factors for cardiotoxicity

CV complications related to G2 were reported in 84 (25.8%) patients, with more than one complication in some patients. Cardiotoxicity was suspected in 49 (15.0%); RVDD in 15 (4.6%); LVDD in 36 (11.0%); and LVSD in 24 (7.4%). There was no statistically significant association between cardiotoxicity and G2 complications (Table 2).

**Table 2:** Association of risk factors for cardiotoxicity with cardiovascular (CV) complications in G2 (ventricular dysfunction)

		CV complication in G2		Total n (%)	p	OR	95% CI
		No n (%)	Yes n (%)				
< 5 years	No	128 (52.89)	54 (64.29)	182 (55.83)	0.070	0.624	0.374 - 1.042
	Yes	114 (47.11)	30 (35.71)	144 (44.17)			
Female sex	Male	141 (58.26)	51 (60.71)	192 (58.90)	0.694	0.903	0.544 - 1.500
	Female	101 (41.74)	33 (39.29)	134 (41.10)			
Genetic syndrome	Yes – Down syndrome	8 (3.31)	1 (1.19)	9 (2.76)	0.561	-	-
	Yes – Others	11 (4.55)	3 (3.57)	14 (4.29)			
	No	223 (92.15)	80 (95.24)	303 (92.94)			
Previous heart disease	Yes	11 (4.55)	3 (3.57)	14 (4.29)	0.947	1.286	0.350 - 4.724
	No	231 (95.45)	81 (96.43)	312 (95.71)			
*CD of anthracyclines	Dose ≤249	181 (74.79)	58 (69.05)	239 (73.31)	0.305	1.330	0.770 - 2.296
	Dose > 250	61 (25.21)	26 (30.95)	87 (26.69)			
Mediastinal / chest	Yes	19 (7.85)	5 (5.95)	24 (7.36)	0.566	1.346	0.486 - 3.726
radiotherapy	No	223 (92.15)	79 (94.05)	302 (92.64)			
Other cardiotoxic drugs	Yes	147 (60.74)	61 (72.62)	208 (63.80)	0.051	0.583	0.338 - 1.006
	No	95 (39.26)	23 (27.38)	118 (36.20)			
**TOTAL**		242 (100)	84 (100)	326 (100)			

CD: cumulative dose; OR: odds ratio; CI: confidence interval.

### Use of cardiovascular medications and dexrazoxane

The most used cardiovascular drugs were diuretics (n=113; 34.7%), followed by antihypertensives (n=56; 17.2%) and vasoactive drugs (n=54;16.6%).

Of 73 patients using dexrazoxane, 18 (24.7%) had G2 complications, and only 36 (49.3%) used cardioprotective agents in 100% of the anthracycline doses. Of the 253 patients that did not use cardioprotective agents for anthracyclines, 66 (26.1%) had G2 complications. The use of dexrazoxane was not significantly associated with cardioprotection (p=0.806; OR=1.078 and [95%CI = 0.591-1.968]).

### CV complications in G1 and G2 and association with age and vital status

CV complications in G1 and G2 occurred in 173 (53.1%) patients. Table 3 summarizes the association of these complications and age at diagnosis, and associations of G1 and G2 complications with vital status are summarized in Table 4.

**Table 3:** Associations of general cardiovascular complications (G1) and ventricular dysfunction (G2) with age at diagnosis

		Faixa etária					Total	p*
		< 1 year n (%)	1-4 years n (%)	5-9 years n (%)	10-14 years n (%)	15-19 years n (%)		
Heart rhythm changes	No	21 (91.3)	109 (90.1)	65 (92.9)	76 (87.4)	23 (92.0)	294 (90.2)	0.839
	Yes	2 (8.7)	12 (9.9)	5 (7.1)	11 (12.6)	2 (8.0)	32 (9.8)	
Myocarditis	No	23 (100.0)	119 (98.3)	69 (98.6)	87 (100.0)	24 (96.0)	322 (98.8)	0.553
	Yes	0 (0.0)	2 (1.7)	1 (1.4)	0 (0.0)	1 (4.0)	4 (1.2)	
Ischemia- AMI	No	23 (100.0)	121 (100.0)	70 (100.0)	87 (100.0)	24 (96.0)	325 (99.7)	0.143
	Yes	0 (0.0)	0 (0.0)	0 (0.0)	0 (0.0)	1 (4.0)	1 (0.3)	
SAH	No	15 (65.2)	104 (85.9)	58 (82.9)	77 (88.5)	22 (88.0)	276 (84.7)	0.080
	Yes	8 (34.8)	17 (14.1)	12 (17.1)	10 (11.5)	3 (12.0)	50 (15.3)	
TEE with catheter	No	23 (100.0)	118 (97.5)	66 (94.3)	83 (95.4)	24 (96.0)	314 (96.3)	0.696
	Yes	0 (0.00)	3 (2.5)	4 (5.7)	4 (4.6)	1 (4.0)	12 (3.7)	
TEE without catheter	No	21 (91.3)	113 (93.4)	63 (90.0)	79 (90.8)	21 (84.0)	297 (91.1)	0.672
	Yes	2 (8.7)	8 (6.6)	7 (10.0)	8 (9.2)	4 (16.0)	29 (8.9)	
PE without anthracyclines	No	22 (95.6)	113 (93.4)	64 (91.4)	82 (94.3)	20 (80.0)	301 (92.3)	0.163
	Yes	1 (4.4)	8 (6.6)	6 (8.6)	5 (5.7)	5 (20.0)	25 (7.7)	
PE with anthracyclines	No	22 (95.6)	112 (92.6)	69 (98.6)	81 (93.1)	23 (92.0)	307 (94.2)	0.481
	Yes	1 (4.4)	9 (7.4)	1 (1.4)	6 (6.9)	2 (8.0)	19 (5.8)	
PE with drainage	No	22 (95.6)	121 (100.0)	70 (100.0)	84 (96.5)	25 (100.0)	322 (98.8)	0.073
	Yes	1 (4.4)	0 (0.0)	0 (0.0)	3 (3.5)	0 (0.0)	4 (1.2)	
CVA	No	22 (95.6)	121 (100.0)	70 (100.0)	87 (100.0)	25 (100.0)	325 (99.7)	0.069
	Yes	1 (4.5)	0 (0.0)	0 (0.0)	0 (0.0)	0 (0.0)	1 (0.3)	
CHF with VAD	No	17 (73.9)	113 (93.4)	66 (94.3)	82 (94.3)	23 (92.0)	301 (92.3)	0.019
	Yes	6 (26.1)	8 (6.6)	4 (5.7)	5 (5.7)	2 (8.0)	25 (7.7)	
Endocarditis	No	22 (95.6)	120 (99.2)	69 (98.6)	86 (98.8)	25 (100.0)	322 (98.8)	0.740
	Yes	1 (4.4)	1 (0.8)	1 (1.4)	1 (1.2)	0 (0.0)	4 (1.2)	
RVDD	No	22 (95.6)	117 (96.7)	68 (97.1)	81 (93.1)	23 (92.0)	311 (95.4)	0.622
	Yes	1 (4.4)	4 (3.3)	2 (2.9)	6 (6.9)	2 (8.0)	15 (4.6)	
Suspected cardiotoxicity	No	19 (82.6)	108 (89.3)	56 (80.0)	74 (85.1)	20 (80.0)	277 (85.0)	0.458
	Yes	4 (17.4)	13 (10.7)	14 (20.0)	13 (14.9)	5 (20.0)	49 (15.0)	
LVDD	No	20 (87.0)	110 (90.9)	63 (90.0)	74 (85.1)	23 (92.0)	290 (89.0)	
	Yes CD=0	1 (4.3)	3 (2.5)	0 (0.0)	6 (6.9)	1 (4.0)	11 (3.4)	0.766
	Yes CD<250	2 (8.7)	6 (5.0)	6 (8.6)	6 (6.9)	1 (4.0)	21 (6.4)	
	Yes CD>250	0 (0.0)	2 (1.6)	1 (1.4)	1 (1.1)	0 (0.0)	4 (1.2)	
LVSD	No	23 (100.0)	118 (97.5)	63 (90.0)	78 (89.6)	20 (80.0)	302 (92.7)	
	Yes CD=0	0 (00.0)	0 (00.00)	0 (00.0)	4 (4.6)	0 (00.0)	4 (1.2)	0.009
	Yes CD<250	0 (00.0)	1 (0.8)	6 (8.6)	4 (4.6)	3 (12.0)	14 (4.3)	
	Yes CD>250	0 (0.0)	2 (1.7)	1 (1.4)	1 (1.2)	2 (8.0)	6 (1.8)	
**TOTAL**		23 (100)	121 (100)	70 (100)	87 (100)	25 (100)	326 (100)	

AMI: acute myocardial infarction; SAH: systemic arterial hypertension; TEE: thromboembolic events; PE: pericardial effusion; CVA: cerebrovascular accident; CHF: congestive heart failure; VAD: vasoactive drugs; RVDD: right ventricular diastolic dysfunction; LVDD: left ventricular diastolic dysfunction; LVSD: left ventricular systolic dysfunction; CD: cumulative dose;

* level of significance of 5%.

**Table 4:** Association of general cardiovascular complications (G1) and ventricular dysfunction (G2) with vital status

		Vital status		Total n (%)	p*	OR	95%CI
		Alive n (%)	Dead n (%)				
Heart rhythm changes	No	210 (89.36)	84 (92.31)	294 (90.18)	0.423	0.700	0.292 - 1.680
	Yes	25 (10.64)	7 (7.69)	32 (9.82)			
Myocarditis	No	234 (99.57)	88 (96.70)	322 (98.77)	0.121	7.977	0.819 - 77.711
	Yes	1 (0.43)	3 (3.30)	4 (1.23)			
Ischemia- AMI	No	235 (100.00)	90 (98.90)	325 (99.69)	0.622	-	-
	Yes	0 (0.00)	1 (1.10)	1 (0.31)			
SAH	No	210 (89.36)	66 (72.53)	276 (84.66)	<0.001	3.182	1.712 - 5.912
	Yes	25 (10.64)	25 (27.47)	50 (15.34)			
TEE with catheter	No	227 (96.60)	87 (95.60)	314 (96.32)	0.921	1.305	0.383 - 4.443
	Sim	8 (3.40)	4 (4.40)	12 (3.68)			
TEE without catheter	No	216 (91.91)	81 (89.01)	297 (91.10)	0.409	1.404	0.626 - 3.146
	Sim	19 (8.09)	10 (10.99)	29 (8.90)			
PE without anthracyclines	No	220 (93.62)	81 (89.01)	301 (92.33)	0.161	1.811	0.782 - 4.193
	Yes	15 (6.38)	10 (10.99)	25 (7.67)			
PE with anthracyclines	No	226 (96.17)	81 (89.01)	307 (94.17)	0.013	3.100	1.216 - 7.902
	Yes	9 (3.83)	10 (10.99)	19 (5.83)			
PE with drainage	No	234 (99.57)	88 (96.70)	322 (98.77)	0.121	7.977	0.819 - 77.711
	Yes	1 (0.43)	3 (3.30)	4 (1.23)			
CVA	No	234 (99.57)	91 (100.00)	325 (99.69)	1.000	-	-
	Yes	1 (0.43)	0 (0.00)	1 (0.31)			
CHF with VAD	No	225 (95.74)	76 (83.52)	301 (92.33)	<0.001	4.441	1.915 - 10.300
	Yes	10 (4.26)	15 (16.48)	25 (7.67)			
Endocarditis	No	232 (98.72)	90 (98.90)	322 (98.77)	1.000	0.859	0.088 - 8.369
	Yes	3 (1.28)	1 (1.10)	4 (1.23)			
LVDD	No	227 (96.60)	84 (92.31)	311 (95.40)	0.173	2.365	0.832 - 6.722
	Yes	8 (3.40)	7 (7.69)	15 (4.60)			
Subclinical cardiotoxicity	No	195 (82.98)	82 (90.11)	277 (84.97)	0.106	0.535	0.248 - 1.153
	Yes	40 (17.02)	9 (9.89)	49 (15.03)			
LVDD	No	211 (89.79)	79 (86.81)	290 (88.96)	0.676	-	-
	Yes CD=0	6 (2.55)	5 (5.49)	11 (3.37)			
	Yes CD<250	15 (6.38)	6 (6.59)	21 (6.44)			
	Yes CD>250	3 (1.28)	1 (1.10)	4 (1.23)			
LVSD	No	219 (93.19)	83 (91.21)	302 (92.64)	0.757	-	-
	Yes CD=0	3 (1.28)	1 (1.10)	4 (1.23)			
	Yes CD<250	10 (4.26)	4 (4.40)	14 (4.29)			
	Yes CD>250	3 (1.28)	3 (3.30)	6 (1.84)			
**TOTAL**	235 (100)	91 (100)	326 (100)				

AMI: acute myocardial infarction; SAH: systemic arterial hypertension; TEE: thromboembolic events; PE: pericardial effusion; CVA: cerebrovascular accident; CHF: congestive heart failure; VAD: vasoactive drugs; RVDD: right ventricular diastolic dysfunction; LVDD: left ventricular diastolic dysfunction; LVSD: left ventricular systolic dysfunction; CD: cumulative dose;

* level of significance of 5%.

### Causes of death

Ninety-one (27.9%) patients died; mean time to death was 17 months, and the causes of death were disease recurrence or progression in 57 (62.6%) patients, infectious and/or parasitic disease in 16 (17.6%), acute respiratory failure in eight (8.8%), CV disease in five (5.5%), and others in five (5.5%).

### Cardiovascular assessment

A total of 216 pediatric patients underwent cardiac assessment and 318 underwent echocardiography (8=97.5%). Figures 1A and B show the percentage of patients that attended the first visit and those who underwent echocardiography before the first dose of anthracycline.

**Figure 1:** A) First visit x first dose of anthracyclines. B) First echocardiogram x First dose of anthracyclines.

## Discussion

Anthracyclines have been used in more than 50% of children and adolescents being treated for cancer.^12^

According to the Ministry of Health, there are 317 centers for the treatment of cancer in Brazil.^13^ Nearly 50% of our patients did not live in the Federal District.

In accordance with the literature, there was a predominance of children of male sex, and younger than four years old (Table 1).^14–16^ In addition, leukemia was the most common neoplasm, corresponding to 33% of all cancers in the age range from 0 to 14 years.^17,18^

During and after treatment, several CV complications may occur, with different clinical presentations and severity^3,10,19^ (Tables 3 and 4), even before the use of anthracyclines in leukemias.^20,21^

According to the literature, during the treatment of cancer, CV abnormalities are the most common complications unrelated to the tumor, that may contribute to early morbidity and mortality in adulthood. Despite that, there is a lack of robust statistical data on the incidence of these complications in this population in Brazil, suggesting that these changes are not even present in the country. Our study showed, in line with the literature, that CV complications were common, but, to our surprise, the most prevalent were those unrelated to ventricular dysfunction. This finding indicates that this form of presentation should be considered in the formulation of preventive strategies.

Tables 3 and 4 describe the frequency of CV complications. SAH was the most prevalent and probably related to the use of glucocorticoids in the induction phase, which may explain why the most used drugs were diuretics and anti-hypertensive medications.^22^ Pericardial effusion may occur in up to 21% of patients;^4^ in the present study, this was the second most common CV complication, occurring even before the use of anthracyclines. Venous TEE were the third most common CV complication and was probably caused by the presence of long-term venous access or by the thrombogenic effect of tumor cells, with an incidence of up to 20% in in-hospital adult patients and 8% in children.^3–5^ The incidence of abnormal heart rhythm may be underestimated due to the non-performance of routine diagnostic methods; the incidence described in the literature is 38%.^4^ CHF due to other causes in patients using vasoactive drugs was the fifth most common complication and may be caused by fluid overload, which favors the development of infectious diseases, with transient myocardial dysfunction and/or septic shock.^23^

Subclinical myocardial injury may occur in the presence of normal LVEF and FS%, which if altered, the damage would be irreversible.^5,24–27^ In search for more sensitive methods, new techniques have been used, including global longitudinal strain (GLS) for the diagnosis of subclinical dysfunction with high sensitivity.^5,24–27^

In the present study, CV complications occurred in more than 50% of patients, and it is known that two out of three survivors may have late CV complications 30 years after cancer treatment.^4,28^

In the analysis of associations of risk factors with CV complications of G2 (Table 2), a CD > 250mg/m^2^ did not show statistical significance, although this was the main risk factor for cardiotoxicity of this group^3,29^ and almost two-thirds of patients had used a CD < 250mg/m^2^. CV complications were not uncommon in G2 (Tables 3 and 4), indicating that there is no safe dose, and subclinical changes were evidenced in the echocardiogram with doses of anthracyclines 100mg/m^2^.^2^

A cardioprotective effect of dexrazoxane as compared with control was demonstrated in controlled studies in which the drug was used prior to the administration of all doses of anthracyclines.^2,6,7^ In our observations, the use of dexrazoxane did not show statistical significance for cardioprotection, which may be explained by the fact that more than 50% of patients did not use the cardioprotective agent prior to all doses of anthracyclines as recommended, which was a bias of our study.^30^

As described in Table 3, CHF with vasoactive drugs showed a significant association with age less than one year, which may be explained by immaturity of the CV system and relative sensitivity of younger cells to chemotherapy.^4^ Also, LVSD was significantly associated with age less than 15 years, which may be due to the prevalence of tumors requiring high CD of anthracyclines.^17^ In Table 4, complications that showed significant association with death – SAH, pericardial effusion after initiation of anthracycline treatment, and CHF with vasoactive drugs – however, in accordance with literature, disease recurrence and progression was the main disease cause of death.^1^

CV complications are the most common complications related to antineoplastic treatment. Cardiac assessment since early stages of treatment, the use of follow-up protocols and preventive measures are of utmost importance.^3,5^ However, nearly 80% of patients underwent echocardiography before starting anthracycline, differently from the visits for cardiac assessment (Figure 1).

This study has limitations that should be considered. The retrospective and single-center design could cause information bias and lack of control of confounding variables (lack of information). Thus, information about CV complications may have been underestimated with the use of M-mode echocardiography (Teichholz formula), since it has been recommended the volumetric assessment of the left ventricle instead (Simpson's biplane method). In the study period, the lack of other methods that may help in the diagnosis of subclinical changes of ventricular function, such as echocardiography with strain imaging, assessment of biomarkers of myocardial injury, as well as routine echocardiography may have underestimated the incidence of G2 complications and abnormal cardiac rhythm.

## Conclusions

Although CV complications related to ventricular dysfunction are the most severe, the most feared and the most studied ones, the present study showed that general complications are the most prevalent. This highlights the need to include these manifestations in the monitoring and preventive strategies.

High CD of anthracyclines is the main risk factor for cardiotoxicity related to ventricular dysfunction, but there is no safe dose. This study reinforces this knowledge, since although 73.3% of the patients used CD < 250mg/m^2^, one out of four patients had these complications.

Despite its limitations and based on the scarcity of published data on CV abnormalities in Brazilian children and adolescents undergoing chemotherapy treatment, this study makes a preliminary report on the subject. However, the clinical scenario here presented certainly reproduces the reality of other cancer centers. Thus, we hope to draw attention to the need for local identification of real demands, focusing on the development of strategies for the enhancement of strengths and correction of deficiencies.

Our findings highlight the importance of a partnership between oncologists and cardiologists in the development of strategies for prevention, diagnosis, optimal early therapy of different cardiotoxicity presentations. This would enable treatment continuity, better quality of life in the future, and reduction of morbidity and mortality rates.
